# Skeletal morphology and phylogenetic relationships of the Late Cretaceous freshwater ray *Myledaphus bipartitus* (Chondrichthyes; Batomorphii)

**DOI:** 10.1080/14772019.2026.2679622

**Published:** 2026-06-25

**Authors:** Julia Türtscher, Patrick L. Jambura, Jürgen Kriwet

**Affiliations:** a University of Vienna, Faculty of Earth Sciences, Geography and Astronomy, Department of Palaeontology, Evolutionary Research Group, Josef-Holaubek-Platz 2, 1090 Vienna, Austria; b University of Vienna, Vienna Doctoral School of Ecology and Evolution (VDSEE), Djerassiplatz 1, 1030 Vienna, Austria; c Johannes Kepler University Linz, Library, Altenberger Straße 69, 4040 Linz, Austria

**Keywords:** anatomy, Batoidea, Campanian, Chondrichthyes, Mesozoic, systematics

## Abstract

In this study, we present the first comprehensive description of the skeletal morphology of the freshwater-tolerant ray †*Myledaphus bipartitus*, a Late Cretaceous batomorph known from North America. These new data are based on an exceptionally well-preserved specimen from the Campanian of the Dinosaur Park Formation in Alberta, Canada, which provides valuable morphological details previously lacking for this taxon. A detailed morphological analysis was conducted, which revealed a unique combination of plesiomorphic and derived characters. Phylogenetic analyses provide support for the placement of †*M. bipartitus* within the order Rhinopristiformes, a group that includes modern guitarfishes and sawfishes. As the oldest known freshwater-tolerant representative of Rhinopristiformes, †*M. bipartitus* thus offers novel insights into the early ecological diversification of this group. The present study underscores the broader necessity for comprehensive morphological and systematic research on Late Cretaceous batomorphs. Nevertheless, the obtained results demonstrate the value of combining detailed morphological data with rigorous phylogenetic methods to elucidate the evolutionary history of extinct and extant batomorph lineages.

## Introduction

The fossil record of sharks and batomorphs, i.e. rays and skates, is extremely rich, but skewed because their skeletons consist of poorly mineralized cartilage with very limited preservation potential. Consequently, the majority of fossil evidence consists of isolated teeth, while articulated skeletal material remains scarce. This paucity of comprehensive morphological data complicates efforts to resolve phylogenetic relationships, particularly among extinct and extant batomorph lineages. These limitations are particularly apparent when considering the Cretaceous, a crucial period for the evolution of modern sharks and rays, with the first appearance of faunas resembling present-day communities (Kriwet, 2003) and an expansion of several taxa into freshwater ecosystems (Maisey et al., 2004). Despite this importance, however, many Cretaceous assemblages remain poorly described and understood, limiting our understanding of their taxonomic diversity, evolutionary history, and ecological significance. Given the ecological importance of modern rays and skates in aquatic ecosystems (Flowers et al., 2021), understanding their deeptime evolution is essential for reconstructing the origins of modern batomorph diversity.

†*Myledaphus bipartitus* Cope, 1876, is a batomorph known from Late Cretaceous deposits of North America and is commonly interpreted as a durophagous, i.e. feeding on hard-shelled prey, freshwater taxon (Hoganson & Murphy, 2002; Wilson et al., 2013; Wroblewski, 2004). The taxonomic classification of the genus and species was originally formulated on the basis of isolated teeth from the upper Campanian Judith River Formation in Montana, USA (Cope, 1876; Kirkland et al., 2013), which are generally relatively common in Late Cretaceous fresh-water and marginal marine deposits throughout North America (e.g. Brinkman, 1990; Case, 1987). However, following its initial description, there has been considerable controversy over the systematic placement of †*Myledaphus bipartitus*, with different authors assigning it to various higher-level taxa. It has been placed in the order Myliobatiformes, family Dasyatidae (based on the shape of its teeth; Brinkman, 1990; Case, 1987; Estes, 1964; Wroblewski, 2004), and in the order Rajiformes (Becker et al., 2004; Hoganson & Murphy, 2002), including the family Hypsobatidae (Beavan & Russell, 1999) and the superfamily Rhinobatoidea (based on the structural makeup of its teeth; Brand et al., 2022; Brinkman et al., 2005; Cappetta, 1987; Peng, 1997; Peng et al., 2001; Kirkland et al., 2013). This superfamily was later incorporated into the then newly established order Rhinopristiformes by Naylor et al. (2012). Additionally, Neuman and Brinkman (2005) discussed its assignment to the family Platyrhinidae (based on the body shape of the specimen examined here), which, at the time, was considered part of ‘guitarfishes’ but was subsequently placed within Torpediniformes. Despite its extensive taxonomic history, no previous studies have determined its phylogenetic position using a cladistic approach based on rigorous morphological character analysis.

Determining the phylogenetic position of †*Myledaphus bipartitus* within batomorphs is crucial for understanding the evolutionary context of freshwater tolerance, as well as for identifying the frequency and lineage-specific evolution of this trait. In this study, we present the first comprehensive description of the skeletal morphology of †*Myledaphus bipartitus*, based on the exceptionally well-preserved, nearly complete specimen from the Campanian Dinosaur Park Formation of Alberta, Canada. The objective of this study is to elucidate the systematic placement of this taxon by combining detailed morphological data with a rigorous phylogenetic analysis. This will contribute to a more comprehensive understanding of the systematics of Late Cretaceous rays and the evolutionary trajectories that led to euryhaline adaptation.

## Material and methods

### Material

The fossil batomorph specimen, TMP1998.062.0001, described here consists of the right half of a very well-preserved ray on a slab of rock in ventral view, discovered in 1998 in middle to late Campanian (Late Cretaceous) alluvial and estuarine sediments of the Dinosaur Park Formation DPF (Belly River Group, Dinosaur Provincial Park) in Alberta, Canada. The specimen is on display at the Royal Tyrrell Museum of Palaeontology (RTMP).

### Methods

Specimen TMP1998.062.0001 was photographed with an Olympus OMD-EM1 Mark II digital camera positioned orthogonally to avoid doubtful results due to a misaligned angle. The specimen was additionally examined under ultraviolet light following the technique described in Tischlinger and Arratia (2013) for better identification of specific skeletal structures.

To minimize the risk to this unique, skeleton-based specimen, its dorsal side was neither prepared nor examined; it is considered too valuable to undergo further invasive work at this time. Additionally, due to its size, CT scanning was not possible using the available equipment.

### Phylogenetic analyses

†*Myledaphus bipartitus* was included in the data matrix of Türtscher et al. (2025), with character scoring based on the only known articulated specimen, TMP1998.062.0001. The data matrix, comprising 77 taxa (44 extant and 33 fossil taxa; the latter indicated by a dagger [†] preceding the taxonomic name), and 142 morphological characters, was modified using Mesquite 3.81 (Maddison & Maddison, 2023).

A parsimony analysis was performed using TNT 1.6 (Goloboff et al., 2008; Goloboff & Morales, 2023), following the protocol of J. C. D. de Carvalho et al. (2025). The analysis consisted of two rounds of tree searches. In the first round, a New Technology search was employed to identify most parsimonious trees, with all search algorithms enabled (Sectorial Search, Ratchet, Drift, and Tree Fusing). The number of random addition sequences was set to 10,000, while all other parameters were kept at their default settings. The second round utilized the Traditional Search algorithm, examining the most parsimonious trees (MPTs) obtained in the first round and retained in memory. Tree bisection and reconnection (TBR) was used as the branch-swapping method, with all other settings left as default. This second round was intended to more exhaustively sample the set of equally parsimonious trees at the established minimum tree length. The resulting MPTs were used to build the consensus tree. Node support was assessed by bootstrap and jackknife resampling, each based on 1000 replicates using the Traditional Search algorithm with default settings.

A maximum likelihood (ML) analysis was performed using the free online phylogenetic tool W-IQ-TREE (Trifinopoulos et al., 2016; available at http://iqtree.cibiv.univie.ac.at/, accessed 22 January 2024). W-IQ-TREE utilizes a dedicated computer cluster at the University of Vienna and is based on the latest version of IQ-TREE (Nguyen et al., 2015). Morphology was set as the sequence type, and the substitution model was automatically determined by the built-in ModelFinder (Kalyaanamoorthy et al., 2017), which selected the MK+FQ+ASC+G4 model as the best-fitting model for the data. Branch support was calculated from 1000 replicates using ultrafast bootstrapping (Hoang et al., 2018).

A Bayesian inference (BI) analysis was conducted using MrBayes v3.2.7 (Ronquist et al., 2012). The analysis was based on the same morphological data matrix and model settings as in the ML analysis, treating characters as discrete and unordered. Two independent runs were performed, each consisting of four Markov chains (one cold and three heated), using Metropolis-coupled Markov chain Monte Carlo (MCMC). The analysis was run for 10 million generations, with trees and parameter values sampled every 1000 generations. Convergence between runs was assessed using the average standard deviation of split frequencies (ASDSF) and potential scale reduction factors (PSRF). A burn-in of 25% was discarded prior to summarizing results. Posterior probabilities were calculated from the majority-rule consensus tree obtained from the post-burn-in samples.

### Systematic palaeontology

Class **Chondrichthyes** Huxley, 1880

Subclass **Elasmobranchii** Bonaparte, 1838

Cohort **Euselachii** Hay, 1902

Subcohort **Neoselachii** Compagno, 1977

Superorder **Batomorphii** Cappetta, 1980

Order **Rhinopristiformes** Naylor, Caira, Jensen, Rosana, Straube, & Lakner, 2012

Family ***incertae sedis***

Genus **†*Myledaphus*** Cope, 1876

**Figure 1:**
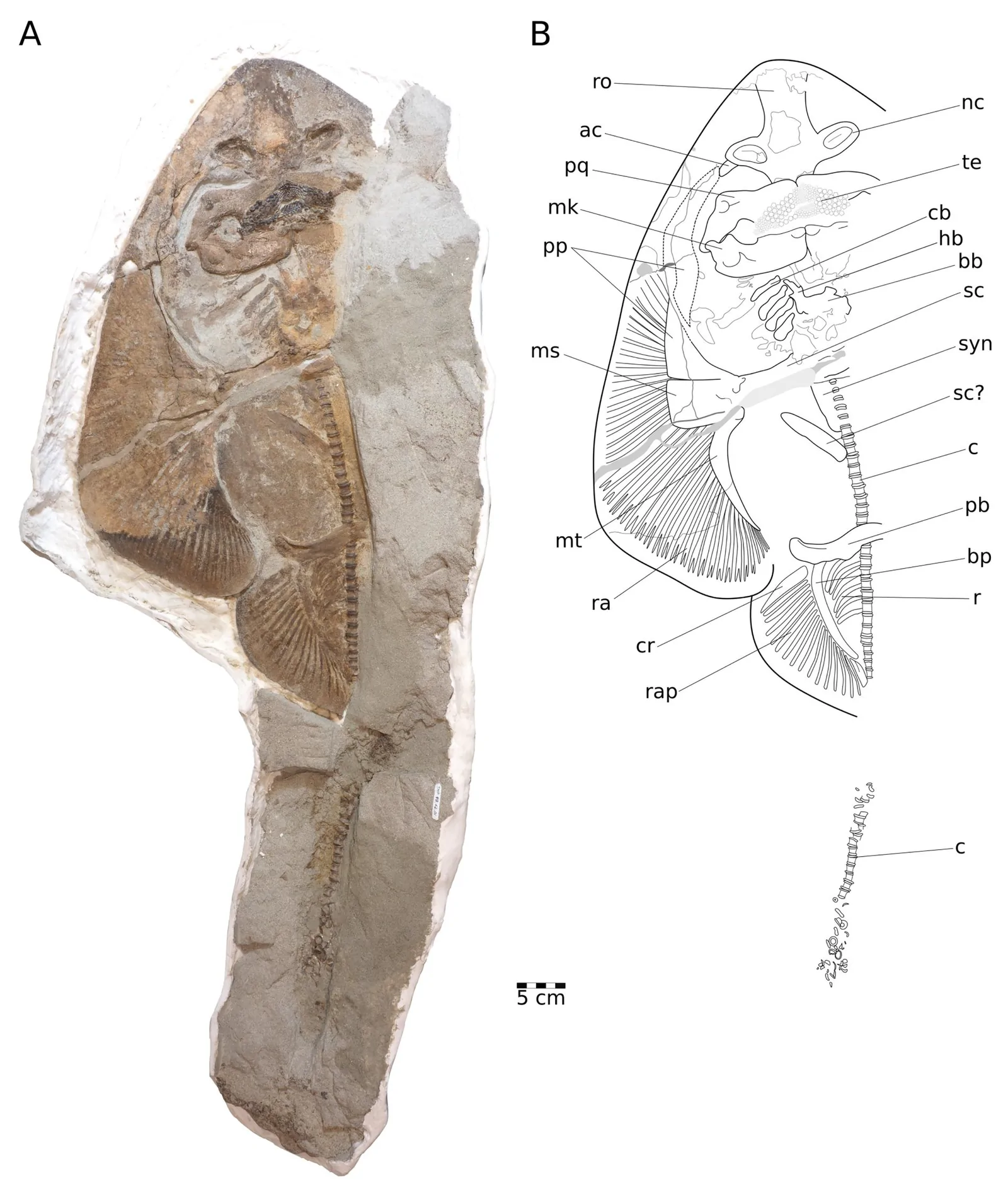
Overview of TMP1998.062.0001, the most complete skeletal specimen of †*Myledaphus bipartitus* known to date. **A**, Photograph of the specimen; **B**, illustration of the specimen showing the skeletal morphology. **Abbreviations: ac**, antorbital cartilage; **bb**, basibranchial; **bp**, basipterygium; **c**, vertebral centra; **cb**, ceratobranchial; **cr**, compound radial; **hb**, hypobranchial; **mk**, Meckel’s cartilage; **ms**, mesopterygium; **mt**, metapterygium; **nc**, nasal capsule; **pb**, puboischiadic bar; **pp**, propterygium; **pq**, palatoquadrate; **r**, ribs; **ra**, pectoral fin radials; **rap**, pelvic fin radials; **ro**, rostrum; **sc**, scapulocoracoid; **syn**, synarcual; **te**, teeth. The scale bar equals 5 cm.

**Figure 2:**
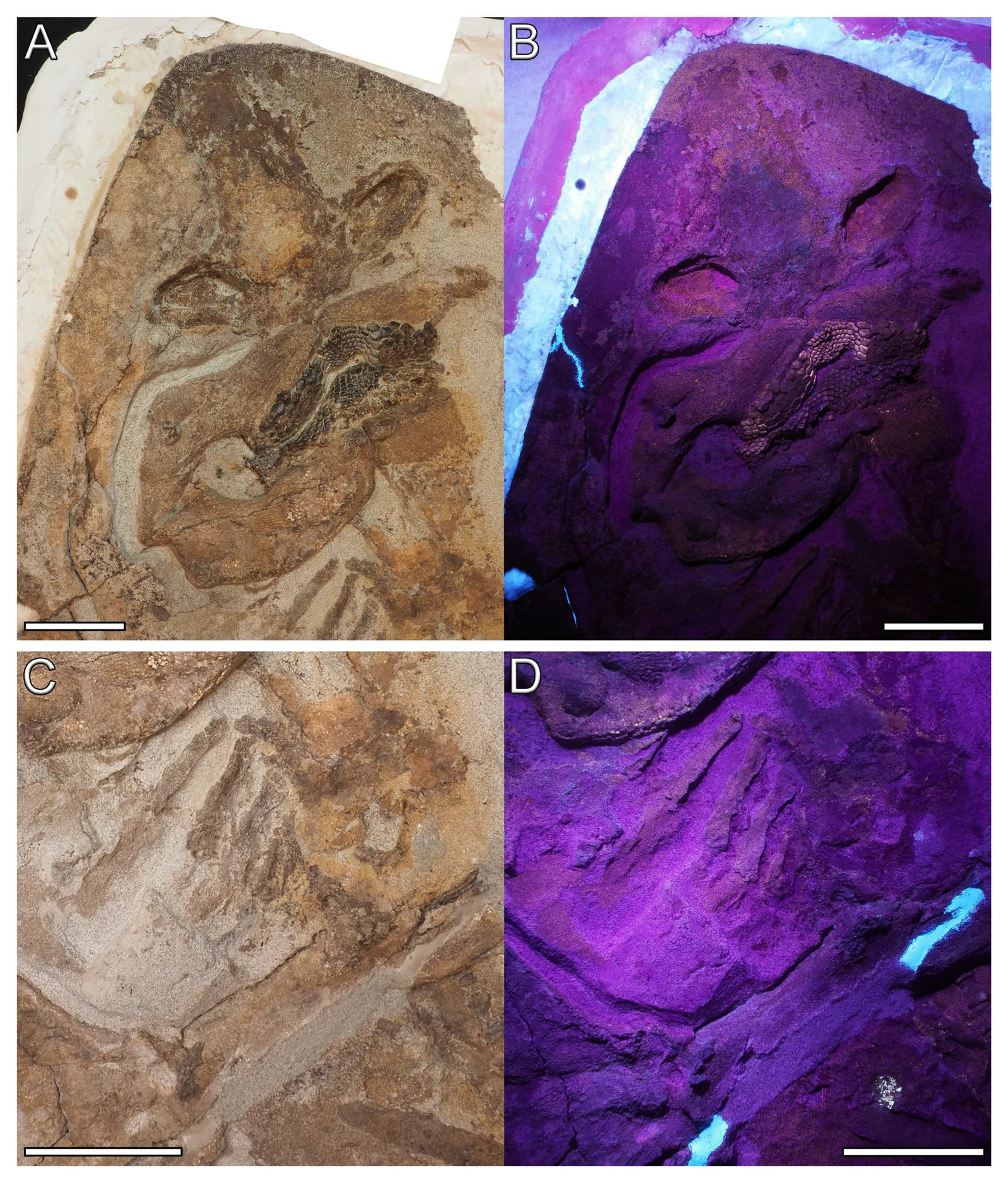
Head- and branchial region of TMP1998.062.0001. **A**, Photograph of the head region of the specimen; **B**, photograph under UV light of the head region of the specimen; **C**, photograph of the branchial region; **D**, photograph under UV light of the branchial region. Scale bars equal 5 cm.

**Figure 3:**
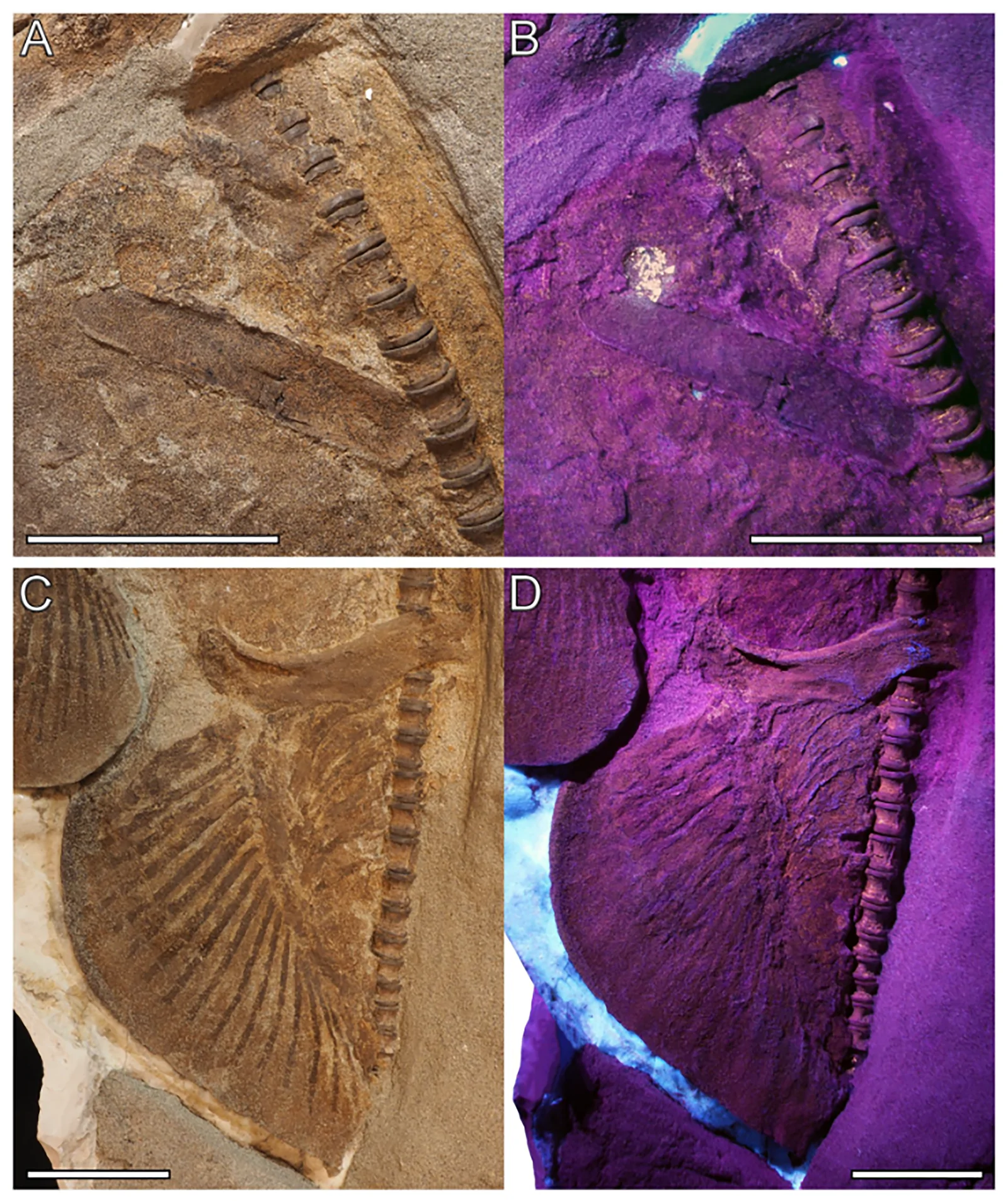
Trunk region of TMP1998.062.0001. **A**, Photograph of the synarcual and possibly a piece of the scapulocoracoid broken off during the taphonomic processes of the specimen; **B**, photograph under UV light of the synarcual and scapulocoracoid piece(?) of the specimen; **C**, photograph of the pelvic region; **D**, photograph under UV light of the pelvic region. Scale bars equal 5 cm.

**Figure 4:**
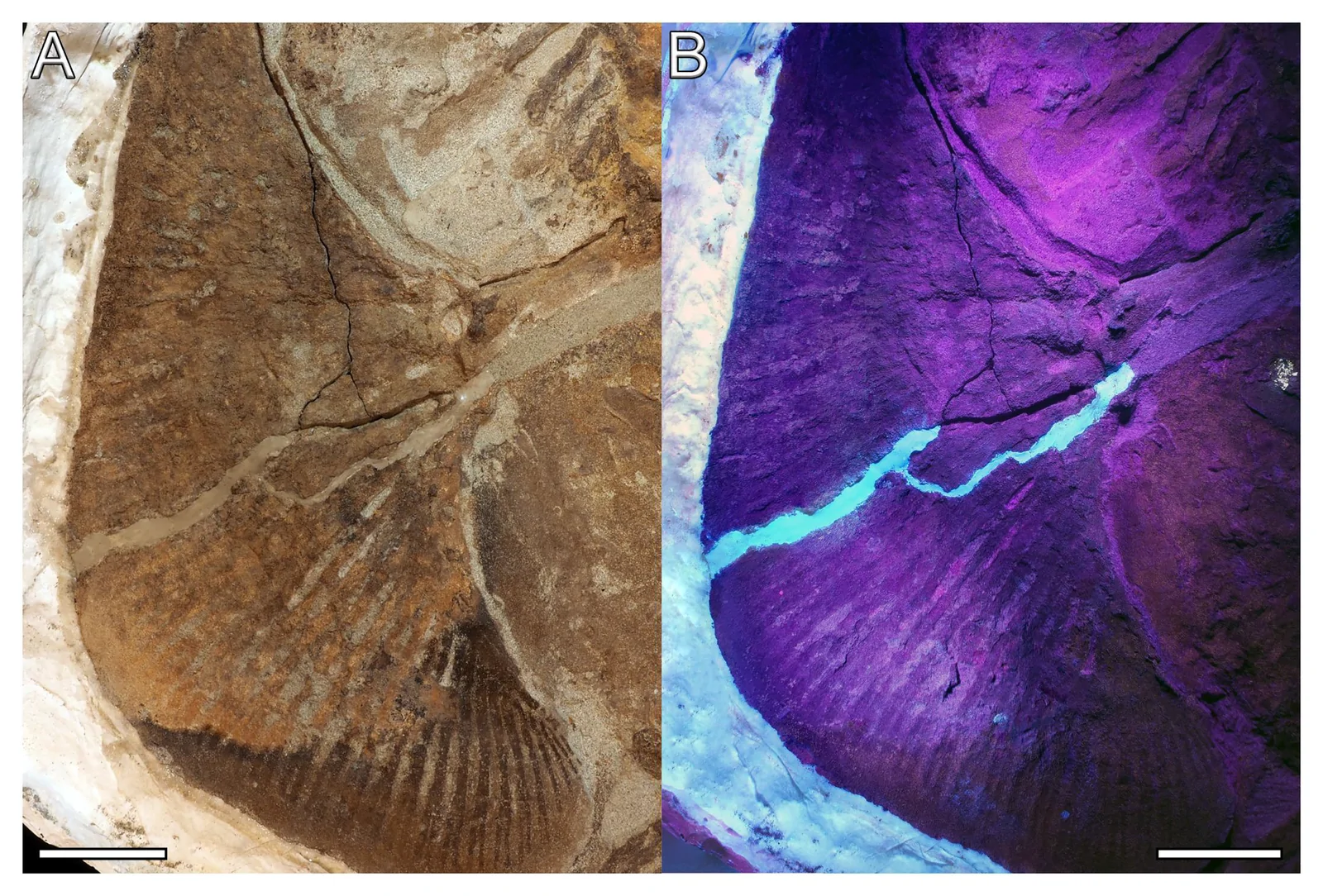
Pectoral fin of TMP1998.062.0001. **A**, Photograph of the pectoral fin of the specimen; **B**, photograph under UV light of the pectoral fin. Scale bars equal 5 cm.

**Figure 5:**
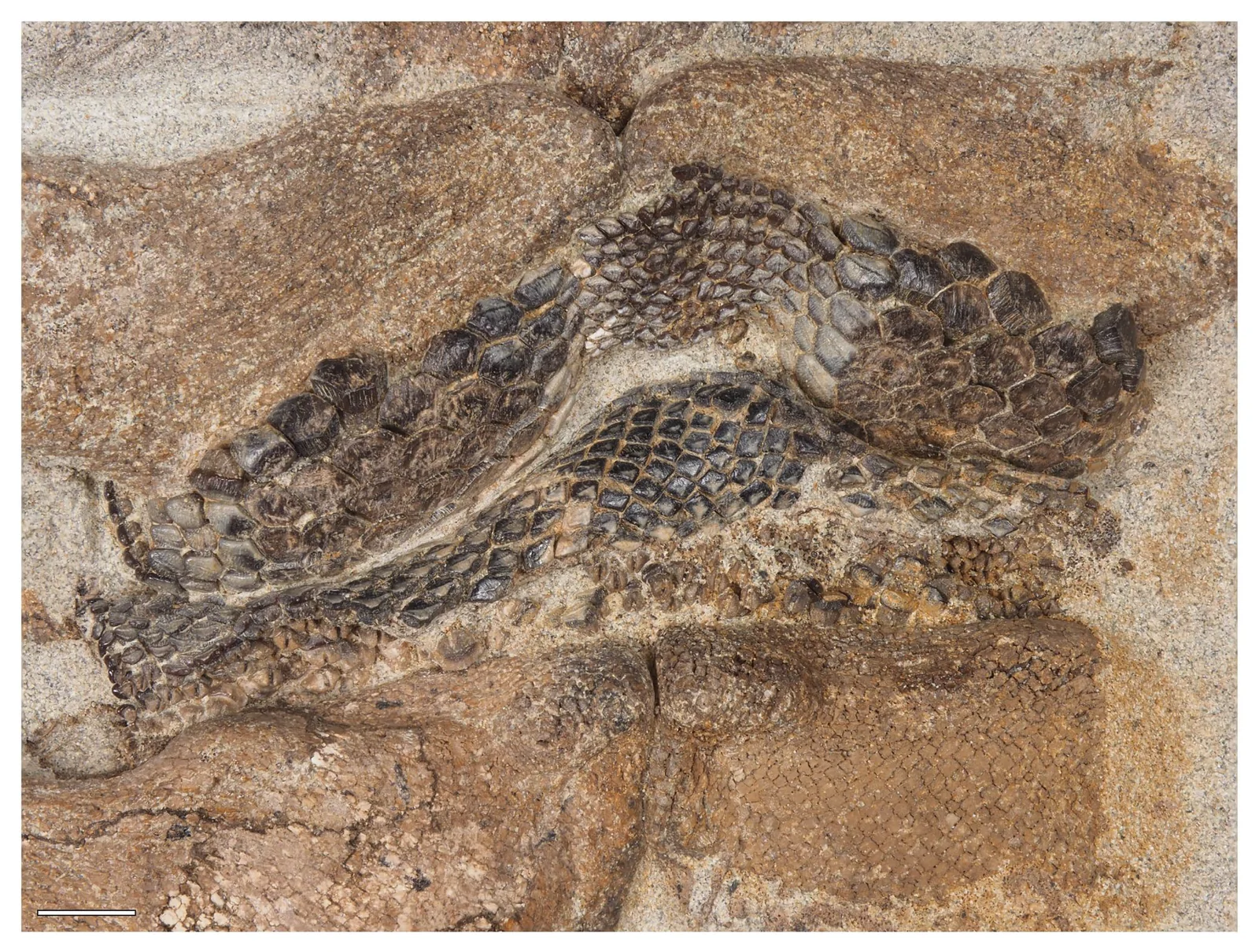
Photograph of the tooth-bearing region on the jaw cartilages of TMP1998.062.0001. Scale bar equals 1 cm.

Species **†*Myledaphus bipartitus*** Cope, 1876 (Figs 1, 2, 3, 4, 5)

2005 *Myledaphus bipartitus* Cope, 1876: Neuman and Brinkman: 170, fig. 9.2

2015 *Myledaphus bipartus* Cope, 1876: Gardner et al.: xii, fig. 3A

#### Type species

†*Myledaphus bipartitus*.

#### Holotype

AMNH FF 7523 (three teeth) from the upper Campanian Judith River Formation, Montana, USA.

#### Stratigraphical and geographical range

Uppermost Cretaceous (Campanian–Maastrichtian) of New Mexico, Colorado, Utah, Wyoming, South Dakota, North Dakota, and Montana in the United States, Alberta and Saskatchewan in Canada, and Mexico (see Brand et al., 2022; Wilson et al., 2013).

#### Material

TMP1998.062.0001, right half of an otherwise almost complete female skeleton in ventral view.

#### Locality, horizon, and age

Canada, Alberta, Dinosaur Provincial Park, Dinosaur Park Formation (DPF), Late Cretaceous, middle to late Campanian. The specimen originates from alluvial to estuarine deposits of the DPF, thus from non-marine to marginal marine conditions.

#### Diagnosis (amended)

A batomorph characterized by a relatively short and broad rostrum with a slight widening at the tip and a rostral appendix. Nasal capsules laterally expanded, oval, and narrower than the jaw cartilages. Antorbital cartilages triangular and posteriorly directed. Palatoquadrate and Meckel’s cartilage massive, equal in anteroposterior depth, with a large tooth-bearing area. Ceratohyal absent. Synarcual elongated and trapezoidal, comprising six vertebral centra. Pectoral fins tribasal and plesodic, with all radials articulating with the basal cartilages; no inter-radial connections. Propterygium triangular, extending anteriorly to the level of the nasal capsule; mesopterygium broad and scoop-like; metapterygium crescent-shaped. Pelvic girdle with fused puboischiadic bar, broad lateral prepelvic processes, and well-developed postpelvic processes. Teeth arranged in a pavement-like pattern; symphyseal teeth rhomboidal, distal teeth hexagonal; crown with parallel enameloid folds and a transverse ridge; root bilobed with a deep median furrow.

### Description

#### Body shape

Medium-sized ray preserved in ventral view; the exact length of the specimen is not known because the tail outline is not preserved, but the length from the tip of the snout to the last preserved caudal vertebral centra measures about 101 cm. The specimen is not entirely preserved but lacks large parts of the left body side; however, it is apparent that the body is rhomboid-shaped. The head is rounded without a strongly elongated rostrum (Fig. 1).

#### Neurocranium

Specimen TMP1998.062.0001 is exposed in ventral view, hence most neurocranial structures are obscured by the cartilages of the upper and lower jaws, i.e. the entire orbital and occipital region. The rostrum is shorter than the rest of the neurocranium; it is rather broad along its entire length and widens slightly at its tip. A rostral appendix is present on the anterior tip of the rostrum. Rostral processes are absent. The nasal capsules are laterally expanded, oval in shape, and inclined anteriorly. They are noticeably less wide than the jaw cartilages. Horn-like processes on the anterior margins of the nasal capsules are absent. The antorbital cartilages are not very well preserved, but it is evident that they are triangular in shape and articulated at the posterolateral margin of the nasal capsules. They are posteriorly directed and short (Figs 1 and 2).

#### Jaw and branchial skeleton

The palatoquadrate and Meckel’s cartilage are relatively equal in anteroposterior depth. They are massive, exhibit prominent processes, and possess a large tooth-bearing area. The jaw cartilages are considerably wider than the nasal capsules. No labial cartilages are present. A ceratohyal is not discernible and therefore considered to be reduced. The basihyal is not preserved. The branchial apparatus is overall not well preserved; however, four to possibly five ceratobranchials are noticeable. Hypobranchial 2 is preserved and appears to be fused with hypobranchial 3–4. The basibranchial is present but only partly preserved (Figs 1 and 2).

#### Axial skeleton and unpaired fins

Primary calcification is evident in all vertebral centra; however, the presence or absence of secondary calcification cannot be determined as none of the vertebrae are broken to reveal their internal structure. There are 16 vertebral centra, including the hemicentrum, between the pectoral and pelvic girdle. Thirteen vertebral centra are present caudally to the pelvic girdle. Eleven articulated caudal vertebral centra are preserved, along with several disarticulated ones. Thus, a total of 40 articulated vertebral centra are preserved. The vertebral centra reach the posterior side of the suprascapula but are not discernible anterior of the suprascapula. A total of six vertebral centra are identifiable in the synarcual. The synarcual is elongated and tapers in width caudally, giving it a trapezoidal shape. Six (pairs of) ribs are present in the pelvic region. The tail outline as well as the dorsal and caudal fins are not preserved. No fin spines are detectable (Figs 1 and 3).

#### Pectoral girdle and fins

The scapulocoracoid seems to be rather broad and straight but it is broken and lacks parts of the coracoid bar. However, in the region between the pectoral and pelvic girdle is an elongated piece of cartilage that appears to have broken off and most likely belongs to the pectoral girdle. The scapular processes are not preserved. The pectoral fins are tribasal and plesodic. All pectoral fin radials articulate with the three basal cartilages; none articulate directly with the fused scapulocoracoid. No inter-radial connections (cross-branches) are present. The propterygium is triangular in shape and extends anteriorly. Parts of the anterior propterygium are broken, but it appears to extend to the level of the nasal capsule. Approximately 12 radials articulate with the propterygium. The mesopterygium is broad and scoop-like and articulates with approximately 10 radials. The crescent-shaped metapterygium articulates with 19 radials. The pectoral fins slightly overlap the pelvic fins (Figs 1 and 4).

#### Pelvic girdle and fins

More than half of the puboischiadic bar is preserved and it is apparent that it is fused. It is rather straight, apart from an anterior curvature in its midpoint. It features broad and triangular lateral prepelvic processes and well-developed postpelvic processes. The basipterygium is elongated and slender. The anterior compound radial is slightly expanded distally and therefore bar-shaped. It is followed by at least 15 radials. Adjacent to the 15th radial is a structure that is not clearly identifiable but may represent a distal piece of the basipterygium that broke off during the taphonomic process. Because of this structure, it is not unambiguously evident if two additional radials are present, which would increase the total number of radials to 17 (in addition to the compound radial). Specimen TMP1998.062.0001 lacks claspers and is thus identified as a female specimen (Figs 1 and 3).

#### Teeth

The teeth are pavement-like and arranged in multiple rows in both the palatoquadrate and Meckel’s cartilage. At the level of the symphysis in both jaws, the teeth are arranged in up to nine rows. Further distally, the teeth increase in size and are arranged in up to six rows. In the palatoquadrate, the teeth are small, rhombus-shaped and arranged in 10–12 files at the level of the symphysis. Further distally, as the teeth increase in size, they are arranged in 10–12 files on the right and left jaw rami and develop a regular hexagonal outline. The increase in size from mesial to distal teeth is less prominent in the Meckel’s cartilage than in the palatoquadrate. The crown exhibits parallel, labiolingually oriented enameloid folds and wrinkles. The oral face is almost flat and has a transverse ridge. The roots are rather high and are separated into two lobes by a deep furrow; they therefore exhibit holaulacorhize root vascularization (see Cappetta, 1987). In a basal view, each of the two root lobes is triangular in shape (see Figs 1 and 5).

### Phylogenetic analysis

**Figure 6:**
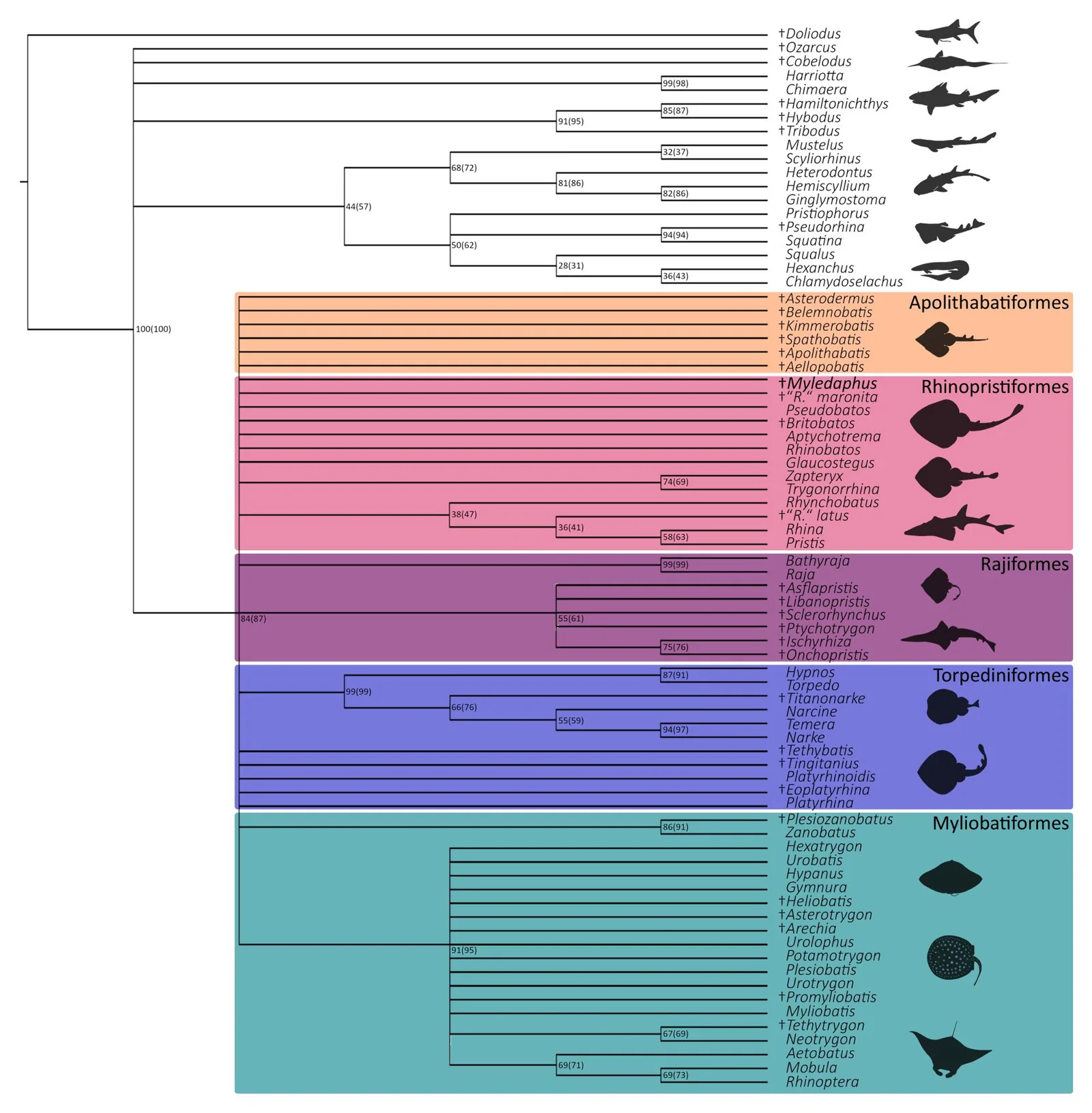
Strict consensus tree with bootstrap and jackknife frequencies (jackknife values in parentheses). Consistency index = 0.427 and retention index = 0.819. Daggers [†] before taxon names indicate extinct taxa. Silhouettes were either drawn by the authors (JT, PLJ) or downloaded from www.phylopic.org (all downloaded images were available for re-use under the Public Domain Dedication 1.0 licence).

The initial phylogenetic analysis, conducted using maximum parsimony New Technology search with all search algorithms enabled (Sectorial Search, Ratchet, Drift and Tree Fusing), yielded 537 most parsimonious trees (MPTs) with a tree length of 375 steps (CI = 0.427 and RI = 0.819). The second round, performed using the Traditional Search algorithm and the trees retained from the initial analysis, resulted in a total of 107,316 MPTs (Supplementary Information: myleda-phus-newtechnology_search.out). †*Myledaphus bipartitus* falls within one of these polytomies and, under the strict consensus, cannot be attributed to any higher clade, i.e., order with certainty (Fig. 6; Supplementary Information: myledaphus-newtechnology_search.out).

**Figure 7:**
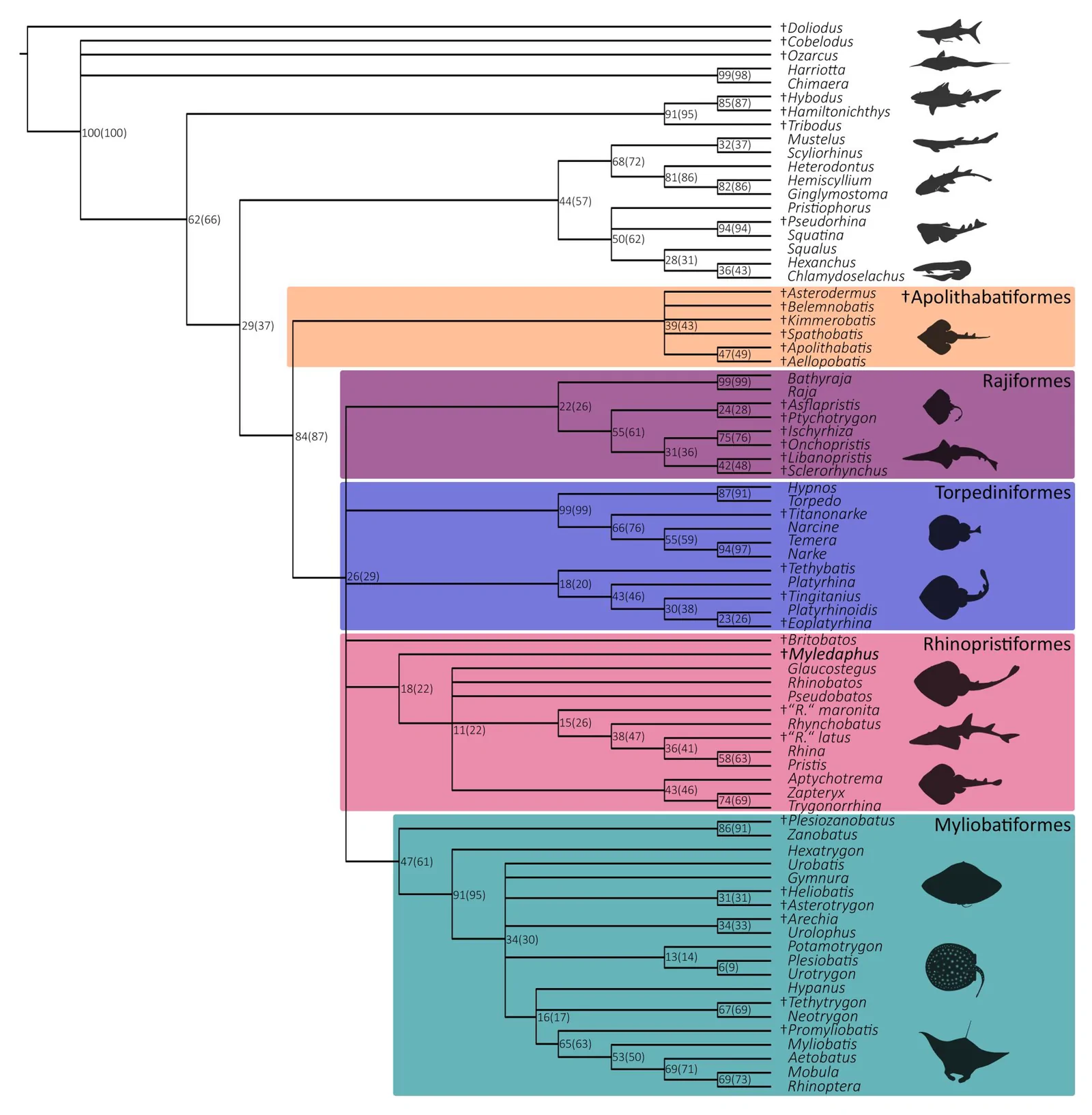
Majority-rule consensus tree with bootstrap and jackknife frequencies (jackknife values in parentheses). Consistency index = 0.427 and retention index = 0.819. Daggers [†] before taxon names indicate extinct taxa. Silhouettes were either drawn by the authors (JT, PLJ) or downloaded from www.phylopic.org (all downloaded images were available for re-use under the Public Domain Dedication 1.0 licence).

Within the majority-rule consensus tree of the maximum parsimony analysis, five monophyletic clades were recovered within crown-group batomorphs: (1) Rajiformes+Sclerorhynchoidei, (2) Torpediniformes (without Platyrhinidae), (3) Platyrhinidae, (4) Rhinopristiformes, and (5) Myliobatiformes (Fig. 7). The interrelationships between these clades remain unresolved. The phylogenetic placement of the fossil guitarfish †*Britobatos* within the crown-group batomorphs remains unresolved as well. This polytomy is resolved in the maximum likelihood and Bayesian inference tree, but here the alleged rhinopristiform taxon †*Britobatos* is placed at the base of the order Myliobatiformes.

†*Myledaphus bipartitus* is placed at the base of Rhinopristiformes in the majority-rule consensus tree. However, the node support is low, which is also the case for the interrelationships of other Rhinopristiformes (Fig. 7).

**Figure 8:**
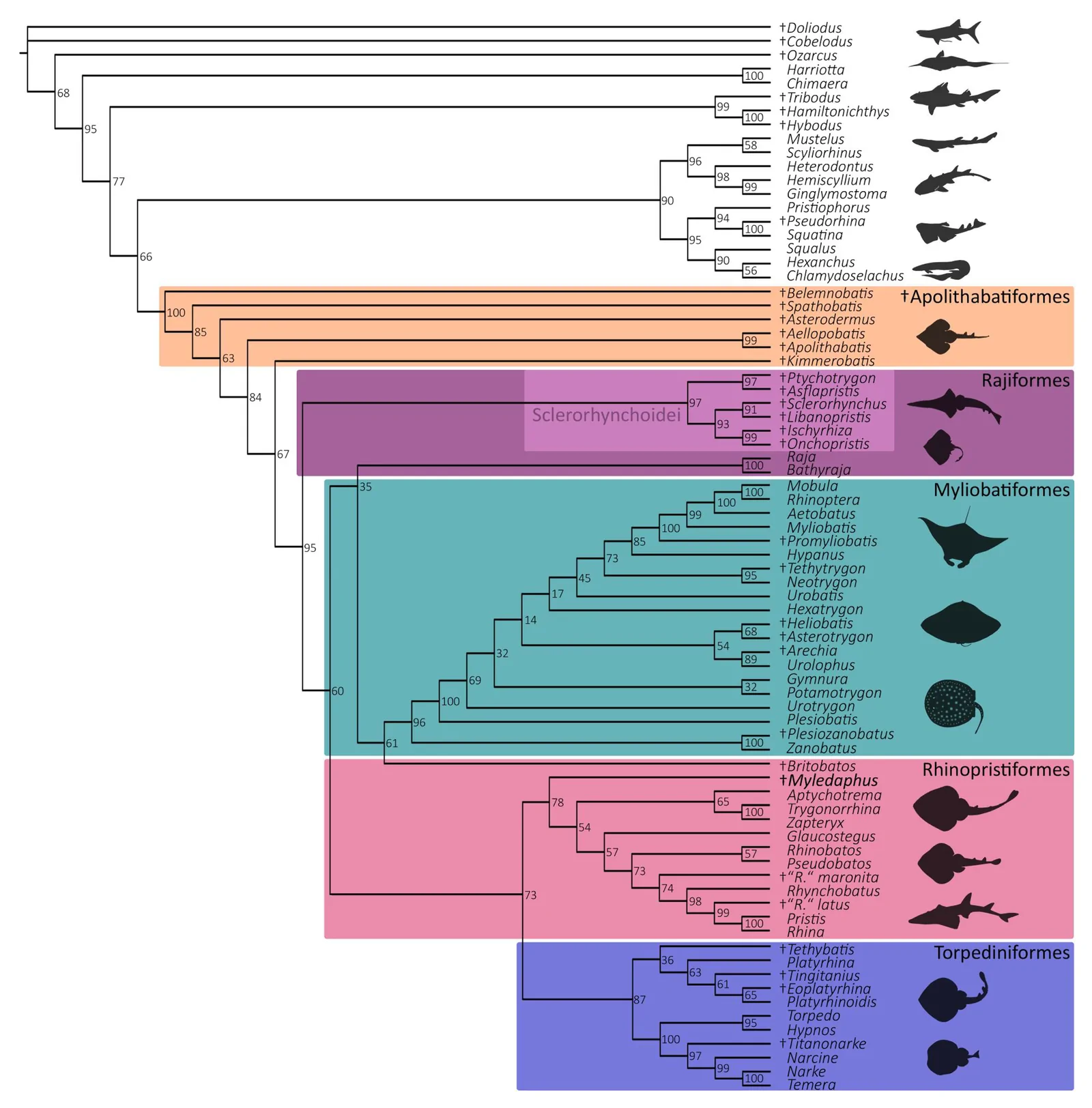
Maximum-likelihood tree with bootstrap frequencies. Daggers [†] before taxon names indicate extinct taxa. Silhouettes were either drawn by the authors (JT, PLJ) or downloaded from www.phylopic.org (all downloaded images were available for re-use under the Public Domain Dedication 1.0 licence).

The maximum likelihood analysis is generally better resolved. †Apolithabatiformes is at the base of the batomorphs, but is not resolved as monophyletic group, similar to previous analyses (Türtscher et al., 2025). Likewise, Rajiformes is also resolved as a polyphyletic group, with the two taxa *Raja* and *Bathyraja* plotted as sister group to Myliobatiformes and not as sister group to the suborder Sclerorhynchoidei. In this tree, Rhinopristiformes is sister to Torpediniformes and †*Britobatos* is not recovered as a rhinopristiform but as a basal myliobatiform. †*Myledaphus bipartitus* is placed as a basal rhinopristiform with moderate node support (bootstrap = 78) (Fig. 8).

**Figure 9:**
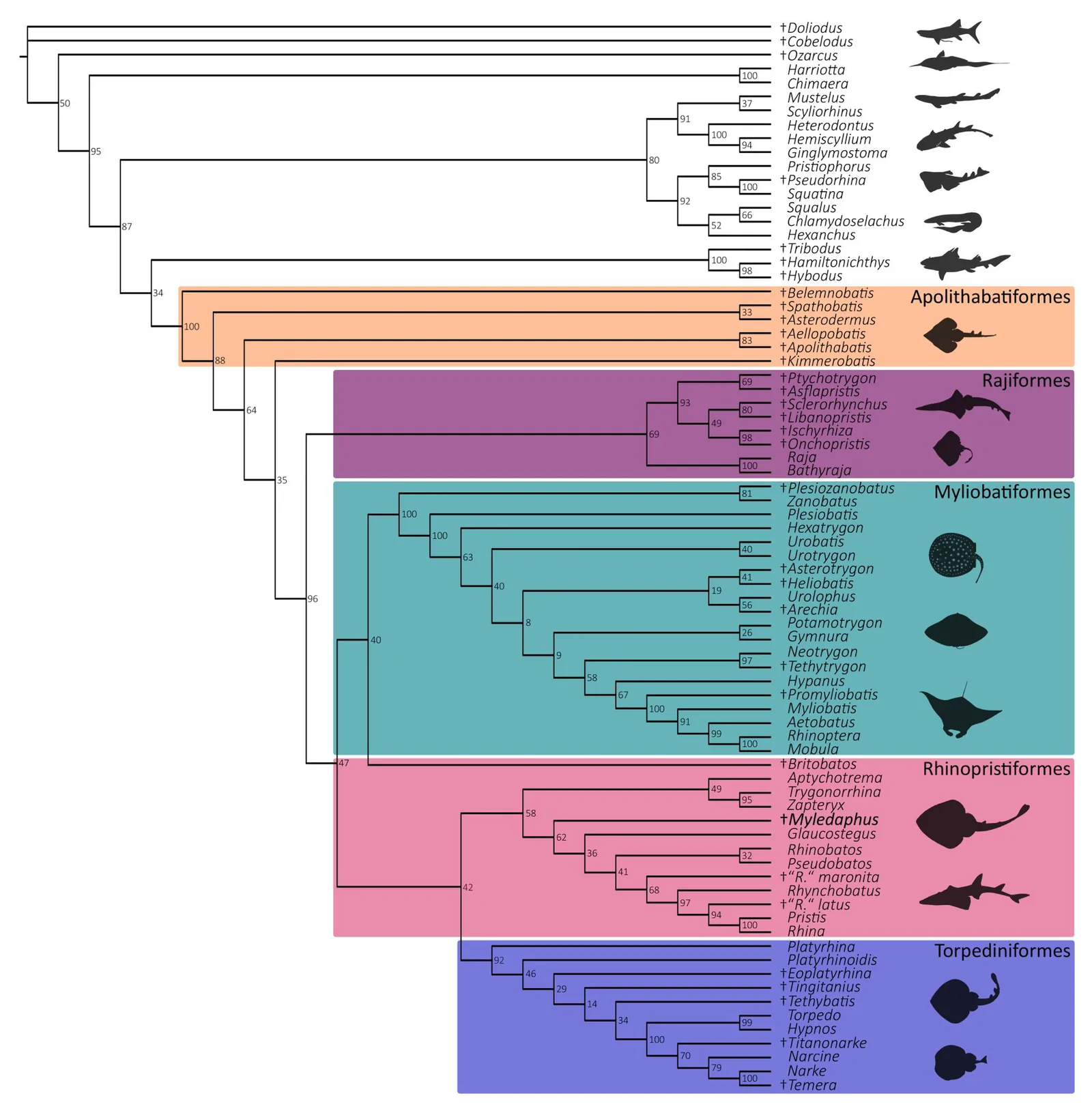
Bayesian inference tree with posterior probabilities. Daggers [†] before taxon names indicate extinct taxa. Silhouettes were either drawn by the authors (JT, PLJ) or downloaded from www.phylopic.org (all downloaded images were available for re-use under the Public Domain Dedication 1.0 licence).

In the Bayesian inference tree, †Apolithabatiformes is likewise not recovered as a monophyletic group. As in the maximum-likelihood tree, †*Britobatos* is placed as a basal myliobatiform, and Rhinopristiformes is sister to Torpediniformes. In contrast to the ML result, Rajiformes is recovered as monophyletic together with †Sclerorhynchoidei at the base of crown-group batomorphs. In this tree, †*Myledaphus bipartitus* is placed at the base of a clade comprising Rhinobatidae, Rhinidae, and Pristidae, with Trygonidae forming the sister group to this assemblage; this relationship is supported by a posterior probability of 0.62 (Fig. 9).

## Discussion

†*Myledaphus bipartitus* is well known from tooth finds of North America (Beavan & Russell, 1999; Becker et al., 2004; Brand et al., 2022; Brinkman et al., 2005; Case, 1978; Kirkland et al., 2013; Peng et al., 2001; Wroblewski, 2004), with TMP1998.062.0001 representing the first nearly complete skeleton of the genus and species. The specimen was first illustrated and briefly described by Neuman and Brinkman (2005), who also discussed a possible assignment to ‘guitarfishes’. Another partial skeleton (consisting of a neurocranium, synarcual and vertebrae) from the Late Cretaceous of western Canada was described by Langston (1970), but it does not preserve any teeth, and it remains uncertain at this time whether or not it belongs to †*Myledaphus*. Therefore, the present study is the first to describe the skeletal features of †*Myledaphus bipartitus* in detail and to place the taxon in a phylogenetic framework. †*Myledaphus bipartitus* is placed at the base of the order Rhinopristiformes in both the majority-rule consensus (Fig. 7) and the maximum likelihood trees (Fig. 8). In the Bayesian inference tree (Fig. 9), it is also located within the Rhinopristiformes, albeit not at the base. Only the strict consensus (Fig. 6) leaves †*M. bipartitus* as unresolved and not assignable to any order with confidence. However, the strict consensus tree is generally highly unresolved, with extensive polytomies among both stem- and crown-group batomorph orders. This is particularly evident in Late Jurassic rays (†Apolithabatiformes *sensu* Türtscher et al., 2025) and Rhinopristiformes, where phylogenetic relationships remain largely ambiguous. Other orders, such as Rajiformes and Torpediniformes, show only partial resolution. This pattern is not unexpected, as the strict consensus retains only those clades present in all maximum parsimony trees (MPTs), collapsing conflicting signals into polytomies. The large number of MPTs recovered (107,316) results in a substantial loss of resolution. Consequently, †*Myledaphus bipartitus* cannot be assigned to any order with confidence under the strict consensus (Fig. 6).

Despite this overall support for its inclusion within Rhinopristiformes, its allocation to a family remains unresolved. In the past, several authors have proposed different systematic placements for †*Myledaphus bipartitus* at the order, superfamily and family level, i.e. Order Myliobatiformes, Family Dasyatidae (Brinkman, 1990; Case, 1978; 1987; Estes, 1964); Order Rajiformes (Becker et al., 2004; Hoganson & Murphy, 2002); Order Rajiformes, Family Hypsobatidae (Beavan & Russell, 1999); Order Rajiformes, Superfamily Rhinobatoidea [proposed by Naylor et al. (2012) to be included in their then newly established order Rhinopristiformes] (Brand et al., 2022; Brinkman et al., 2005; Cappetta, 1987; Kirkland et al., 2013; Peng, 1997; Peng et al., 2001); Order Torpediniformes, Family Platyrhinidae [note that Platyrhinidae was considered to be ‘guitarfishes’ at the time and only later were they included in Torpediniformes] (Neuman & Brinkman, 2005). In the following, we consider several morphological characters (some of which are synapomorphies for different systematic units), discuss whether they support or reject the assignment of †*Myledaphus bipartitus* to each unit, and argue why we are confident in our result of placing it within Rhinopristiformes but without further systematic assignment (numbers in square brackets indicate the numbering of the states):

### Rostral processes

The presence of rostral processes [1] is a synapomorphy of Torpediniformes [see maximum parsimony tree (MP) (character 7) in Villalobos-Segura et al. (2022)]. All other batomorphs, including †*Myledaphus bipartitus*, lack rostral processes [0].

### Rostral appendix

In Myliobatiformes, a rostral appendix is either absent [0] or its presence/absence is currently unknown [?]. Torpediniforms also lack a rostral appendix. The absence of a rostral appendix is a synapomorphy of a clade comprising Torpediniformes and Myliobatiformes [see maximum likelihood tree (ML) (character 9) in Villalobos-Segura et al. (2022)]. On the other hand, the presence of a rostral appendix [1] is regarded as a synapomorphy for Rhinopristiformes [see MP in Villalobos-Segura et al. (2022)], as well as independently gained by crown-group Rajiformes and most †Apolithabatiformes. A rostral appendix is also present in †*M. bipartitus*, thus not supporting a myliobatiform or torpediniform attribution but a rhinopristiform assignment.

### Antorbital cartilages with regular outline

The presence of small or reduced antorbital cartilages [1] is a synapomorphy for Myliobatiformes [see MP and ML (character 25) in Villalobos-Segura et al. (2022)]. The antorbital cartilages in †*M. bipartitus* are well developed [0] and therefore do not support its placement within Myliobatiformes.

### Position of the articulation of the antorbital cartilage on nasal capsule

In most batomorphs including myliobatiforms the articulation of the antorbital cartilage on the nasal capsule is lateral [0]. An anterolateral articulation [1] is considered a synapomorphy for the torpediniform clade that includes *Hypnos, Narcine, Narke, Temera, Torpedo* and †*Titanonarke* [see MP and ML (character 110) in Villalobos-Segura et al. (2022)]. The posterolateral articulation between the antorbital cartilage and the nasal capsule [2] is a synapomorphy of the rhinopristiform clade, which includes *Pseudobatos, Rhinobatos, Glaucostegus*, †‘*R*.’ *maronita*, †’*R*.’ *latus, Rhynchobatus, Rhina* and *Pristis* [see MP and ML in Villalobos-Segura et al. (2022)]. In †*Myledaphus bipartitus*, the articulation is also posterolateral, supporting its assignment to Rhinopristiformes.

### Hypobranchial–basibranchial articulation

The absence of an articulation surface between the basibranchial and hypobranchial [1] is a synapomorphy of Rajiformes [see MP and ML (character 42) in Villalobos-Segura et al. (2022)]. Such an articulation surface is also absent in several myliobatiform taxa. In †Apolithabatiformes, Rhinopristiformes, and Torpediniformes, the hypobranchial articulates with the basibranchial [0], which is also the case in †*Myledaphus bipartitus*.

### First segment of the propterygium

In Myliobatiformes except Zanobatidae, the first segment of the propterygium either reaches the level of the nasal capsules [1] or extends well beyond the nasal capsules [2] [see MP and ML (character 108) in Villalobos-Segura et al. (2022)]. The basal state for batomorphs is the first segmentation of the propterygium not reaching the nasal capsules [0], which is also the case for †*M. bipartitus* and thus separates it from derived Myliobatiformes.

### Proximal propterygium position relative to procondyle

A posteriorly to the procondyle extending proximal portion of the propterygium [1] represents a synapomorphy for Myliobatiformes, but also an independent gain for the torpediniform *Platyrhina* and the rhinopristiform †*Britobatos* [see MP and ML (character 112) in Villalobos-Segura et al. (2022)]. †*Myledaphus bipartitus* differs from Myliobatiformes in that the proximal part of the propterygium does not extend beyond the procondyle [0].

### Postpelvic processes

The presence of postpelvic processes [1] is a synapomorphy in the parsimony tree reconstruction of Villalobos-Segura et al. (2022: character 118) for torpediniforms, with a secondary loss in *Narke* and *Temera*. In general, postpelvic processes are absent [0] in most batomorphs, but are present in most torpediniforms, some rhinopristiform taxa, and the apolithabatiform taxon †*Spathobatis bugesiacus*. †*Myledaphus bipartitus* is also among those taxa that have postpelvic processes, distinguishing it in particular from Myliobatiformes and Rajiformes.

### Anterior margin of puboischiadic bar

A straight anterior margin of the puboischiadic bar [0] is the basal state for batomorphs. An anteriorly arched anterior margin of the puboischiadic bar [1], conversely, is a synapomorphy for Myliobatiformes, with secondary losses in †*Asterotrygon* and †*Heliobatis* [see MP and ML (character 120) in Villalobos-Segura et al. (2022)]. Arching of the anterior puboischiadic bar margin is also present in some apolithabatiforms and rhinopristiforms, as well as in †*M. bipartitus*. While †*M. bipartitus* thus shares this character state with most myliobatiforms and some rhinopristiforms, it clearly separates it from torpediniforms.

### Position of vertebral centra in the synarcual relative to position of suprascapula in synarcual

The vertebral centra in the synarcual reaching caudally to the suprascapula [2] is the basal state for batomorphs, as it is present in several taxa across all orders except in †Apolithabatiformes. In †Apolithabatiformes and the torpediniform *Platyrhinoidis*, vertebral centra are present along the entire length of the synarcual [0]. A synarcual with vertebral centra that reach anterior to the suprascapula [1] is a shared feature of the torpediniform taxa *Narke* and *Temera*, as well as several rhinopristiform taxa [see MP (character 55) in Villalobos-Segura et al. (2022)]. †*Myledaphus bipartitus* also has a synarcual with vertebral centra reaching anteriorly to the suprascapula, further supporting its placement in Rhinopristiformes.

### Vertebral ribs

The presence of vertebral ribs [1] is the basal state for batomorphs. The absence of vertebral ribs [0] is a synapomorphy for the clade that includes all myliobatiforms except all zanobatids, as well as the two rajiforms *Bathyraja* and *Raja* (see MP and ML (character 90) in Villalobos-Segura et al. (2022)). †*Myledaphus bipartitus* has multiple pairs of vertebral ribs, which clearly separates it from Myliobatiformes.

### Second synarcual

All myliobatiforms except zanobatids share the presence of a second synarcual [1] as a synapomorphy [see MP and ML (character 88) in Villalobos-Segura et al. (2022)]. The absence of a second synarcual [0] is thus the basal state for batomorphs, including †*Myledaphus bipartitus*.

Taken together, a combination of plesiomorphic and derived characters strongly supports the placement of †*Myledaphus bipartitus* in the order Rhinopristiformes. While some characters are shared with other batomorph clades – such as Myliobatiformes and Torpediniformes – these characters are either homoplastic or represent the basal state for Batomorphii. The overall morphological pattern observed in specimen TMP1998.062.0001 is also more consistent with members of the order Rhinopristiformes than with alternative placements proposed in previous studies (e.g. Brinkman, 1990; Case, 1978, 1987; Estes, 1964). This placement thus provides a working framework for interpreting the ecology and evolutionary history of †*Myledaphus bipartitus*, particularly in light of its occurrence in freshwater and marginal marine environments. Recognizing †*Myledaphus bipartitus* as a freshwater-tolerant species indicates that the ability to tolerate freshwater evolved early within Rhinopristiformes, which is consistent with multiple independent origins of freshwater tolerance within this group.

The vast majority of extinct and extant batomorphs are adapted to a life in marine environments, while obligate freshwater taxa and freshwater-tolerant taxa are relatively rare (Compagno, 1990; Lucifora et al., 2017). Present-day freshwater batomorphs are best represented by the subfamily Potamotrygoninae (order Myliobatiformes, family Potamotrygonidae), a diverse and endemic group from South America that includes genera such as the eponymous *Potamotrygon, Paratrygon, Plesiotrygon* and *Heliotrygon* (Last et al., 2016; Lucifora et al., 2017). Beyond this clade, a few euryhaline taxa, such as *Hypanus sabinus* (order Myliobatiformes, family Dasyatidae) in North America and *Urogymnus polylepis* (order Myliobatiformes, family Dasyatidae) in Southeast Asia, have a wider salinity tolerance, as evidenced by their occurrence in freshwater, estuarine and coastal marine habitats (Grant et al., 2019; Last et al., 2016; Snelson & Williams, 1981). Extant guitarfishes such as *Glaucostegus typus* (order Rhinopristiformes, family Glaucostegidae) as well as sawfishes such as *Pristis pristis* (order Rhinopristiformes, family Pristidae) are also known to be freshwater-tolerant, with juveniles in particular occupying estuarine and brackish habitats (Heemstra et al., 2022; Kyne et al., 2021; Last & Stevens, 2009; Last et al., 2016; Thorburn et al., 2007).

The fossil record provides compelling evidence for the repeated and independent evolution of freshwater tolerance among batomorphs. Notable examples include several Cretaceous sclerorhynchoid sawfishes, such as †*Onchopristis* (order Rajiformes, suborder Sclerorhynchoidei), known from fluvial and estuarine deposits, suggesting at least partial adaptation to non-marine environments (Guinot & Cavin, 2016; Villalobos-Segura et al., 2021). From the Eocene, †*Propristis* (order Rhinopristiformes, family Pristidae) is also interpreted to have inhabited marginal-marine or estuarine environments (Farrés & Fierstine, 2009). In the Eocene Green River Formation of North America, the stingrays †*Heliobatis radians* and †*Asterotrygon maloneyi* (order Myliobatiformes, family Dasyatidae) are well documented from exclusively freshwater lake deposits (M. R. de Carvalho et al., 2004). Fossil representatives of modern freshwater stingrays, Potamotrygonidae, are common in Neogene deposits of Middle and South America, with their oldest records occurring in the Eocene of Amazonian Peru (Adnet et al., 2014).

The occurrence of †*Myledaphus* in freshwater and marginal marine environments of the Late Cretaceous of North America (e.g. Kirkland et al., 2013; Neuman & Brinkman, 2005; Wroblewski, 2004) thus represents the earliest known case of freshwater inhabitation within the order Rhinopristiformes. These extinct taxa – together with their extant counterparts – thus illustrate the recurrent and convergent evolution of freshwater tolerance within Batomorphii across geological time. Collectively, these examples suggest that freshwater tolerance evolved independently in batomorphs at least six times throughout their evolutionary history, occurring across multiple lineages, including Sclerorhynchoidei, Pristidae, Myliobatiformes (Potamotrygonidae and Dasyatidae), and Rhinopristiformes.

## Conclusion

The skeletal morphology of †*Myledaphus bipartitus*, described here in detail for the first time, provides important insights into the early evolutionary history of crown-group batomorphs. Specimen TMP1998.062.0001 displays a combination of plesiomorphic and derived characters, which together support a placement within the order Rhinopristiformes. As a potential early fresh-water-tolerant representative of the group, †*M. bipartitus* also serves as a case study for the convergent evolution of euryhalinity in batomorphs. However, despite significant advances in our understanding of batomorph phylogeny, the limited resolution of the current phylogenetic framework underscores how much remains unknown about early diversity and relationships within Rhinopristiformes, e.g. with respect to other Cretaceous taxa such as the Lebanese guitarfishes. A thorough and meticulous re-examination of these still poorly understood fossil taxa is therefore essential to resolve their taxonomy and systematics, and thus to refine our understanding of the evolution of batomorphs during this critical period in their evolutionary history. Nonetheless, the placement of †*Myledaphus bipartitus* within Rhinopristiformes suggests that freshwater tolerance evolved early in this group and evolved independently at least two times within Rhinopristiformes and six times across all batomorphs.

## Supplementary Material

Character list

## Data Availability

All data supporting the results of this study are available through MorphoBank at https://doi.org/10.7934/P6382 (Türtscher et al., 2026). The fossil material presented in this study is held in a publicly accessible collection, details of which are available in the paper.
